# Pivotal Role of Quantum Dots in the Advancement of Healthcare Research

**DOI:** 10.1155/2021/2096208

**Published:** 2021-08-06

**Authors:** Pawan K. Tiwari, Mugdha Sahu, Gagan Kumar, Mohsen Ashourian

**Affiliations:** ^1^Department of Physics, Birla Institute of Technology, Mesra, Ranchi 835215, Jharkhand, India; ^2^Department of Electrical Engineering, Majlesi Branch, Islamic Azad University, Isfahan 8631656451, Iran

## Abstract

The quantum dot is a kind of nanoparticle whose dimension is smaller than the size of a typical nanoparticle ranging from tens of nanometers to a few hundredths of nanometers. The quantum mechanical behavior associated with the quantum dot displays different optical and electronic properties, enabling the quantum dot to find potential applications in a multitude of areas such as solar cells, light-emitting diodes, lasers, and biomedical applications. The objective of this investigation is to explore its fundamentals, synthesis, and applications, especially in the healthcare domain. We have discussed the quantum dot synthesis techniques using chemical methods, namely, wet-chemical methods and vapor-phase methods and plasma processing methods, namely, an ion sputtering method and plasma-enhanced chemical vapor deposition method. We have thoroughly investigated the application of quantum dots in imaging, diagnostics, and gene therapy areas. A significant outcome of this review is to propose quantum dots as a new modality in the treatment of cancer and gene therapeutics in the healthcare domain and the potentials of artificial intelligence to improve their performance via the applications of neural networks.

## 1. Introduction

From solar panels to medical applications, quantum dots are receiving discernible attention in today's world due to their unparalleled and cutting-edge scope [1, 2]. Quantum dots are colloidal semiconductor nanoparticles that exhibit a distinctive set of optical and transport properties due to their spatial confinement regime, also known as the quantum confinement effect. In bulk semiconductors, the presence of multiple atoms causes splitting of electronic energy levels which when grouped forms an energy band. The most filled band, known as the valence band, is at lower energy, and the mostly empty band known as the conduction band is at relatively higher energy. The valence band and conduction band are forbidden by an energy gap, called a bandgap. In order to excite a valence electron to the conduction band, the applied radiation of energy radiation must be equivalent to the forbidden energy of the bandgap. After absorption of suitable energy, an electron (e−) can jump into conduction from the valence band which causes the formation of vacant space in the valence band, known as a hole (h+). This pair of electrons (e−) and hole (h+) can be perceived as a hydrogen-like species and is known as an exciton. These excitons for a specific semiconductor bear a separation between the electron (e−) and hole (h+) which is called the exciton Bohr radius. In quantum dots, the excitons are confined to a much smaller volume of the semiconductor material which is in order of its exciton Bohr radius. This results in less splitting of the energy bands and leads to a quantum confinement region [3]. Such a region of electron-hole pairs in various dimensions within a material and the electronic energy bands associated with it are discrete and quantized. The size and the composition of the quantum dots can be altered to allow the energy levels and the bandgap to be fine-tuned to specific desired energies. Quantum dots are bandgap tunable by their dimension (diameter ranging typically from 2 to 10 nm) which means that their optical and electrical properties can be engineered to meet specific biomedical applications [4].

Nowadays, these quantum dots have been the subject of numerous exceptional reviews. One important attribute to note is that quantum dots are semiconductor nanocrystals. One of the most important characteristics of quantum dots is the conductive region of semiconductor heterostructure that comprises the precisely regulated quantity of excess electrons which are constrained to a small region by the application of external electromagnetic radiations. These parameters can be altered to achieve the desired configuration of the quantum dot to meet the requirements of different applications. The energy levels of quantum dots are mainly determined by two factors, first, the size of the quantum dots, and second, the intensity of external electromagnetic fields under which the corresponding quantum dots are exposed. Moreover, quantum dots can also be used in the field of quantum computation and artificial intelligence (AI). The quantum dots can be fabricated on the layer of semiconductor heterostructure to construct a three-dimensional matrix of quantum registers. The fact that quantum dots can easily be drafted and controlled according to application requirements makes it advantageous, particularly for quantum neural processing networks [5].

The structure of these nanocrystals comprises a fluorescent semiconductor ‘core' which has excellent optical properties (i.e., emission and absorption spectrum) due to coating with another semiconductor material which acts as passivation for “core” and is hence known as “shell”. The shell has a generally greater bandgap than the core shell that passivates the surface of the core and inhibits nonradiative recombination processes, which can act as a trap state of electrons in the conduction band, and as a consequence, it can lead to a reduction in the fluorescence efficiency, also referred to as photobleaching. Thus, the passivation of the core from the shell is important to improve its photostability and increase quantum yield (brightness) and also to reduce chemical attacks. As a result, the optical properties of quantum dots get less sensitive to changes in their surroundings. This characteristic of core-shell quantum dots is widely used in applications for biological imaging, chemical sensing, and optoelectronics [3].

## 2. Fabrication of Quantum Dots

Fabrication of quantum dots can be led by various techniques according to the core requisites of the application. These methods of synthesis of quantum dots can be broadly categorized into two approaches, bottom-up approach and top-down approach [6]. The bottom-up synthesis technique approach includes self-organized processes such as colloidal synthesis, ion sputtering, plasma-assisted epitaxy, and others. Alternatively, the top-down approach techniques include ion implantation, molecular beam epitaxy (MBE), X-ray lithography, and electron-beam lithography [7].

### 2.1. Fabrication Using Various Mechanisms

**Figure 1:**
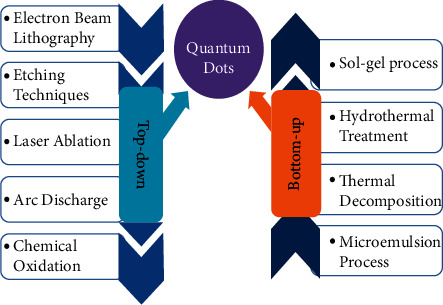
Methods of fabrication of quantum dots.

Figure 1 shows methods of fabrication of quantum dot. A bulk semiconductor is thinned to make quantum dots in the top-down method. Electron-beam lithography, reactive-ion etching, and/or wet-chemical etching are widely used to create quantum dots with a diameter of about 30 nm. For efficient quantum confinement effect tests, optimized dimensions with defined packing geometries are possible. Alternatively, arrays of zero-dimension quantum dots have been created using focused ion or laser beams. These fabrication processes, however, have significant disadvantages. These processes include the integration of impurities into quantum dots and the patterning of structural flaws [8–14].

Another well-known technique, etching, is important in nanoscale fabrication. Dry etching involves embedding a reactive gas species into an etching chamber and applying a radio frequency voltage to create a plasma that breaks down the gas particles into more volatile parts [7]. These high-kinetic-energy species collide with the surface and form an unstable reaction species, which is used to etch a patterned sample [8]. The etching method for ions is known as reactive-ion etching (RIE). The precise etching of the substrate is done using a masking pattern. The fabrication of GaAs/AlGaAs quantum structures as small as 40 nm has been detailed using RIE and a blend of boron trichloride and argon [7, 8].

Various techniques are used to synthesize quantum dots in the bottom-up approach, and they can be narrowly divided into wet-chemical and vapor-phase approaches. Wet-chemical methods such as microemulsion, sol-gel, competitive reaction chemistry, hot-solution decomposition, and electrochemistry are all classified as (1) wet-chemical methods. Vapor-phase methods include self-assembly of nanostructures in material grown by molecular beam epitaxy (MBE), ion sputtering, liquid metal ion sources, and aggregation of gaseous monomers [5].

### 2.2. Wet-Chemical Methods

Wet-chemical methods are generally used to prepare colloidal quantum dots. These methods are based on the conventionally performed precipitation methods with more precisely controlled parameters for single or compounded solutions. Invariably, both homogeneous nucleation (condensation and crystallization) and controlled growth of quantum dots are involved in the precipitation methods. Quantum dots of the desired shape, composition, and dimension can be accomplished by altering, layer thickness, temperature, electrostatic potential, micelle or stabilizers forming, forerunner concentrations, concentrations of anionic and cationic agents, and solvent [5].

However, there are some inherent drawbacks, such as relative instability, associated with the colloidal synthesis of quantum dots. When chemically synthesized quantum dots are coupled with devices, stabilization is mandatory. The stabilization of quantum dots usually involves the construction of a core-shell structure and passivation of quantum dot nanocrystals. Lately, surface chemistry methods of immersing quantum dots in polymer/glass matrices have aroused interest. By this modiﬁcation, the dangling bonds of quantum dots are blocked. In both quantum dots/polymer and quantum dots/glass structures, distinct optical properties have been shown. As a result, they are used in configurable lasers to support fluorescent materials in order to achieve maximum color emissions [5].

### 2.3. Vapor-Phase Methods

The vapor-phase methods of synthesis of nanocrystals (quantum dots) are broadly based on the phenomenon of homogeneous nucleation and deposition of vapor-phase composition and formation of the solid particle which is to be restricted to nanoparticulated size and shape. The fundamental cause for the nucleation and deposition of vapor-phase composition is the thermal unitability of vapor-phase composition which is due to self-generated surrounding conditions. The whole process of synthesis of quantum dots occurs in a special type of chamber, also called process chamber, to alter the surrounding conditions such as temperature and pressure. The topic name ‘vapor-phase syntheses' indicates the involvement of the concept of supersaturated vapor. In the state of supersaturation, the vapor-phase composition is on the verge of transition to condense phase. The process of phase transition may also involve a chemical reaction. Since vapor-phase methods are performed in the process chamber, the state of the supersaturated vapor phase can be generated thermodynamically or by altering pressure [6]. When the level of saturation is high enough for the nucleation vapor phase, particles will nucleate homogeneously through the kinetics of chemical reaction or condensation. Once the molecules or atoms start to nucleate, the remaining vapor-phase molecules get to react chemically or condense on the nucleated particle to relieve the latent supersaturation, and rather than further nucleation, growth of the quantum dots nanocrystal occurs. Therefore, to produce small quantum dots, the degree of supersaturation of the vapor phase must be high. Further, when the growth of quantum dots reaches the desired size, further growth can be obstructed by immediately quenching the system by eliminating the source of supersaturation. In spite of the fact that quantum dots can be self-assembled utilizing vapor-phase techniques to produce quantum dots arrays without a layout, the change within the estimate of quantum dots frequently results in inhomogeneous optoelectronic properties [14, 15].

### 2.4. Plasma Processing of Quantum Dots

**Figure 2:**
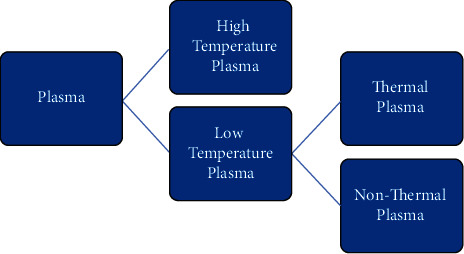
Characterization of plasma.

Figure 2 shows the characterization of plasma. Low-pressure plasmas are an intriguing source for the meticulous preparation of group IV and III–V nanocrystals. Plasma is a quasi-neutral gas made up of a number of dissimilar entities including electrons, positive and negative ions, free radicals, gas atoms, and molecules in their ground or higher states [6]. This ionized gas can occur at a wide range of temperatures and is formed at low pressure or atmospheric pressure and can occur in a wide temperature range. It is achieved by binding energy to a gaseous environment using different methods such as mechanical, thermal, chemical, radiant, nuclear, applying voltage, injecting electromagnetic waves, and a combination of these methods to divide the gaseous constituent particles into a populace of ions, electrons, charge-neutral gas molecules, and other particles. Plasmas are classified into two types: (1) high temperature or fusion plasmas, in which all electrons, ions, and neutral species are in thermal equilibrium, and (2) low-temperature plasmas. Low-temperature plasmas branch out into thermal plasma and are referred to as quasi-equilibrium plasmas. They are in a state of local thermal equilibrium (LTE). Nonthermal plasma (NTP), also known as a nonequilibrium plasma or cold plasma, is another kind of plasma [16].

The wet-chemical synthesis routes in colloidal solutions are well established, according to mainstream investigations in the field of quantum dots conducted on II-VI and IV-VI chalcogenide semiconductors. Group IV semiconductor nanocrystals (NCs), such as Si or Ge, and several III–V semiconductor nanocrystals (NCs), such as GaAs, GaN, and InAs, are difficult to make in solution due to their stronger covalent bonding and therefore higher melting points. As a second route, electron-beam lithography enables the fabrication of structures with nanometer dimensions but has a severe drawback of the low amount of serial lithographic methods. Therefore, the fabrication of well-deﬁned quantum dots must be conducted by various plasma processes. A further benefit of plasma synthesis over liquid phase methods is that the resulting material is devoid of ligands or surfactants. Depending on the application, these ligands may be added later. This makes it easier to integrate plasma-produced NCs into devices because it eliminates the need for the intermediate ligand exchanges that are normally needed for II-VI and IV-VI NCs. Ion sputtering and Plasma-Enhanced Chemical Vapor Deposition (PECVD) are two plasma-assisted techniques for producing quantum dots (QDs) [16, 17].

### 2.5. Ion Sputtering Method

One of the most cost-effective and efficient methods of fabrication of highly dense and homogeneous nanoparticles is the surface sputtering technique, which is broadly a Physical Vapor Deposition (PVD) of bulk semiconductors (cathode). It is a phenomenon in which the semiconductor surface (target material) is bombarded by a beam of energetic particles of plasma, partially ionized gas (commonly Ar). This technique is also known as ion sputtering; this approach involves the utilization of phenomena occurring on the surfaces of bulk semiconductors exposed in the vacuumed chamber to the beam of energetic particles, especially ions. These ions are generated as a spatially clustered beam from gas discharged plasma, and for directed flow, they are accelerated to predetermined energies by the electric field, to have sufficient energy to get projected onto the substrate (Anode). The process of sputtering is commonly performed inside a special device known as an evacuated chamber. For ion beam milling and ion assistance, energy ranging from ∼100 eV to 5 keV is used [16]. The sputtering mechanism involves besieging the solid semiconductor surface with energetic gaseous particles (usually Ar+) which are accelerated under high voltage. These energetic gas particles (plasma) encountered with the solid semiconductor's surface particles or sometimes whole atoms of the semiconductor material are expelled out and impelled toward the target substrate and develop a really strong bond. Mechanical forces firmly bind the ensuing film deposition formed by the projected ions to the substrate (Anode), but in certain situations, an alloy or chemical bond may also be formed.

### 2.6. Plasma-Enhanced Chemical Vapor Deposition (PECVD) Method

Another reliable and renowned synthesis method that has been gaining popularity recently due to its distinctive applications unlike the different stages of microelectronic circuit fabrication is the Plasma-Enhanced Chemical Vapor Deposition (PECVD) method. PECVD is a method for the fabrication of large-scale ICs (integrated circuits) and thin ﬁlm transistors. In Plasma-Enhanced Chemical Vapor Deposition, the reactive gases are decomposed via electrical discharge. This enables ﬁlm deposition at lower temperatures than CVD [16]. In CVD, the substrate is heated to produce thermal energy that is required to control chemical reactions. Thermal energy imparts the energy necessary to break bonds. In PECVD, a plasma is used to decompose the gas molecules for ﬁlm deposition. Thus, plasma is here essentially an energy source replacing thermal energy and can perform the same operations at lower temperatures as conventional processes achieve at elevated temperatures. Therefore, it enables low-temperature processing to be conducted for several synthesis steps. Besides lowering the deposition temperature, PECVD provides superior control over ﬁlm properties through plasma effects. Thus, when opposed to the traditional CVD process, PECVD offers twofold advantages: low processing temperature and the flexibility of film properties. A low processing temperature meets the small thermal expenditure criterion for many manufacturing applications, while the flexibility of film properties allows for customizing properties to achieve the desired device characteristics. Now, Plasma-Enhanced Chemical Vapor Deposition is being used in upcoming applications such as the synthesis of silicon nanocrystals due to its versatility.

## 3. Applications of Quantum Dots in Versatile Medical Domains

Due to their various beneficial properties, such as fluorescence, solvation in water, biocompatibility, low toxicity, compact size and ease of modification, rational scale-up processing, and versatile conjugation with other nanoparticles, quantum dots have risen as a potential material in a variety of biomedical applications. As a result, they have become a popular option for a variety of biomedical applications, including drug nanocarriers, therapeutic genes, photosensitizers, and antibacterial particles. Their capabilities must be proven in multifunctional diagnostic levels, cellular and bacterial bioimaging, and the advancement of theragnostic nanomedicine. They will be used shortly for identifying various types of cancer cells, atomic components of disease, and current drug action mechanisms, applying them to intracellular/extracellular considerations, and developing previously untested biochemical assay strategies.

### 3.1. Diagnostics and Bioimaging

Currently, significant research is aimed to use the distinct optical properties of quantum dots in diagnostics and bioimaging. In the past decades, the most common method of labeling biological entities has been with organic fluorophores. But organic fluorophores have some limitations such as the instability of organic fluorophores when exposed to photoirradiation results in degradation and photobleaching over time. This implies that it does not continuously fluoresce for an extended period of time [18]. Typically, conventional organic fluorophores have a very limited excitation range which allows simultaneous excitation of several organic fluorophores very challenging, resulting in incompatibility in multicolor applications. Dyes are more receptive to their immediate surrounding conditions, such as changes in pH level, causing nonradiative recombination processes [3, 19].

On the other hand, quantum dots have unique optical properties that make them ideal for biological imaging and tagging applications for several reasons [20]. First, quantum dots have a higher extinction coefficient and typically have a broad excitation range and narrow emission peak. This allows multiplex bioimaging with a single source [20]. Second, quantum dots have high quantum yields (QYs), which helps in producing high contrast imaging [18, 21]. Third, they show less photobleaching compared to conventional dyes. Fourth, their size-tunable property of absorption and emission spectrum allows imaging and tagging multiple targets at the same time. Fifth, due to the narrow emission spectrum and minimal interference of the spectrum, quantum dots can be used for the same assay with minimal spectral-overlapping with each other. Sixth, the inorganic properties of quantum dots make them less toxic than conventional organic dyes [22]. Seventh, quantum dots can be functionalized with different bioactive agents to affect the dynamics and functioning of the target biomolecules.

Quantum dots that have a near-infrared (650–900 nm) emission spectrum can be used to prevent any interference from the autofluorescence emitted by cells, hemoglobin, and water. The absorption coefficients and scattering effects of cells, hemoglobin, and water are lower in the near-infrared region. Intravital microscopy is usually performed with light, but imaging deeper tissues necessitate the use of near-infrared light. Moreover, under ultraviolet excitation, quantum dots are more photostable than organic molecules and have more saturated fluorescence [6].

Quantum dots have promising applications in cellular labeling, deep-tissue imaging, and assay labeling due to their unique optical and surface properties, which make them an excellent alternative to conventionally used organic dyes. Besides, their unique optical properties make them excellent fluorescence resonance energy transfer (FRET) donors [21]. FRET is the distance-dependent phenomenon based on the nonradiative transmission of photoexcitation energy from donor to acceptor through dipole-dipole interaction. FRET from quantum dots to organic acceptor molecules allows real-time sensing and recognition of the target [21].

The bioassays and biomedical investigation can be broadly categorized into two parts in vitro and in vivo. In in vitro, bioassays are performed in a controlled and artificial environment whereas, in in vivo, biological systems or biomolecular bioassays are investigated in the presence of living organisms [23]. The selective quantum dots and their purpose of use are categorized into in vivo and in vitro in Tables 1 and 2.

### 3.2. Drug Research

In the last two decades, and even currently, there has been a lot of research focused on targeted and rate-controlled drug delivery. One of the main concerns in medical treatment is the target and rate-controlled drug delivery to lower the side effects of conventional chemotherapy and other treatment methods. The targeting could be by utilizing pH-level maintenance paramagnetic nanoparticle controlling, ultrasonic waves interaction, and temperature control to localize and target the delivery of the drug to the diseased cells [21]. Unlike conventional organic fluorophores, the multivalent nature of quantum dots enables different ligands to attach to the same quantum dot. In addition, the surface chemistry of the quantum dot may confer additional characteristics to the quantum dot. For example, a quantum dot surface can be coated with a paramagnetic substance and can be used in MRI [6, 21].

The surface properties and high surface area of quantum dots make it suitable for conjugation with different kinds of biomolecules in order to localize the drug delivery to the target cell and can also be traced because of the unique optical and electrochemical properties of quantum dots. To reduce the toxicity of therapeutic agents, it is crucial to penetrate through the cell membrane for the targeted delivery of therapeutic agents or drugs. Also, the surface properties of quantum dots allow bioconjugation with some proteins and peptides, which are found to be capable of penetrating cell membranes. Cell-penetrating peptides (CPPs) are the most widely used biomolecules for penetration through the cell membranes and are compatible with most of the therapeutic agents, nucleic acids, proteins, and liposomes [24]. CPPs are used for internalization to cells of quantum dots which comprises therapeutic agents or drugs. Moreover, some studies also show that quantum dots can be internalized to cells without any biofictionalization by optimizing the size, charge, and surface properties of the respective quantum dots [21, 22]. Studies show that the positively charged particle can easily penetrate through the cell membrane, and the larger ones are maintained to the cytoplasm region while smaller ones can penetrate through the nucleus [19]. Once they localize to their respective target, the drug release can be automatically initiated in a pH-controlled manner. Tumor cells have less pH than the corresponding cells [21]. Moreover, drug release can also be triggered externally from electromagnetic radiation which can change the thermal state quantum dot [18]. Quantum dots should be selectively conjugated with biomolecules according to the respective target. Quantum dots can be used as specific biomolecule sensors, like sugar maltose; they can be surface conjugated with protein which has a binding affinity to maltose. Likewise, to target special antigens, quantum dots should be conjugated with an appropriate antibody [21, 24]. The most studied bioconjugation of quantum dots is a surface fictionalization of foliate to target foliate receptors overexpressed in many types of tumor cells [24]. Recent researches have shown the capability of quantum dot for targeted drug delivery and tracing the delivery process of the drugs simultaneously. Encapsulation of quantum dots is crucial to reduce toxicity and to prevent drug release before attaching to the target cell membrane [3, 19]. Thus, quantum dots are encapsulated in biodegradable polymer like the most appropriate polymer to encapsulate anticancer drug which is chitosan [24]. The size-tunable emission spectrum of quantum dots makes it beneficial for simultaneous multicolor imaging of different targets in the body [6, 18, 23].

### 3.3. Gene Therapy

Numerous studies are being conducted by scientists across the globe to modify the genes, carry out gene replacement of the defective ones and novel ways to cure or prevent diseases, and improve health quality. In the past few decades, with the rising need for development in healthcare research in gene therapy, tremendous capabilities have been shown in a variety of applications. Its prominence has been evidently portrayed in several manifestations of developing concepts such as CRISPR-based genome editing and epigenetic research. The growing prominence of researches based on the science of metabolomics, proteomics, and transcriptomics will further expedite the imminent breakthrough of gene therapy [25, 26].

Gene therapy has been quite instrumental in the treatment of numerous health issues, such as cancers or tumors, neurological malfunctions, dermatological disorders, cardiovascular disorders, ophthalmologic disorders, inner ear disorders, and transmittable diseases [27]. However, nowadays, gene therapy is being utilized in a broader and unlimited aspect. Small interfering RNA, antisense oligonucleotides, and microRNA are various other therapeutic nucleic acid materials that have been included within the conventions of quality therapy [25, 28]. Several viral vectors have been proven to be effective in carrying out these tasks. However, due to some poisonous immunological impacts shown by these approaches, quantum dots have been developed as a safe and compelling nonviral gene vector within the domain of gene therapy [25, 29].

Gene therapy necessitates the use of multifunctionalized and biocompatible delivery platforms penetrating through the cell membranes. The optical and surface properties and high biocompatible surface area of quantum dots make it suitable for conjugation with different kinds of fictionalizers, so quantum dots can function as multifuctionalized delivery platforms for gene therapy [25]. Owing to the impressive size-tunable properties of absorption and emission spectrum and high quantum yield, quantum dots act as a competent FRET donor. Quantum dots utilized as delivery platforms are optically traceable and competent in tying different bioligands, such as DNA, RNA, cell-penetrating peptides (CPPs), and nuclear localization groupings (NLS), without aggravating the natural function [30]. Quantum dot-conjugated oligonucleotide arrangements that are joined through surface carboxylic acid groups may be focused on to tie with DNA or mRNA [31, 32]. Various experiments held by scientists revealed that when siRNA is internalized into a cell in conjugation with quantum dots, the synthesis of test protein in the cell gets reduced by 98 percent. Additionally, the significant role of the quantum dots in RNA technology is to detect mRNA molecules by utilizing in situ hybridization (ISH) and also for the combination of siRNA with RNA treatment applications. In the ISH technique, quantum dots are successfully used to examine the expression of unambiguous mRNA transcripts in mouse midbrain regions [33].

## 4. Artificial Intelligence in Quantum Dot Biomedical Applications

Artificial intelligence provides new potentials for biomedical applications such as the prediction of patients' responses to therapies, the early diagnosis of a variety of disorders, and giving specific treatment options to the patients [32–46]. The support vector machine (SVM) learning method provides us with the lesser root mean square errors (RMSE) which is a result of optimization of the weights, *W*, calculated through the expression

(1) $$\begin{array}{c} W=\sum_{j=1}^{N}\left(\alpha_{j}-\alpha_{j}^{*}\right)X_{j}, \end{array}$$

where *α* and *X* are variables [47].

Artificial neural network (ANN) is applied for modeling the relationships between the in vitro data and in vivo effects [48] in asthmatic patients receiving the monodisperse aerosols of salbutamol sulfate.

ANN has been used widely to find cause-and-effect relationships and prediction of in vitro and in vivo data (IVIVC) correlations [47–53]. The ANN helps to find the interpolation of the pharmacokinetic parameters and constructing complex relationships among the constituent parameters [53].

In [54], a multilayer perceptron (MLP) feedforward network has been used for modeling the effects of causal factors on in vitro release profile of theophylline. The authors examined several training algorithms besides the experimental datasets. They found the gradient descent backpropagation algorithms as the best training algorithms for modeling and prediction of drug release profiles [54]. Using ANN results in improvement of coating levels and reducing the amount of chitosan-pectin complex results in the retarded drug release. As compared to another causal factor, coating weight gain, chitosan-pectin in the coating solution played a significantly influential role in determining dissolution profiles [54].

ANN and genetic algorithm are used to predict and optimize in vitro proliferation mineral medium for G × N15 rootstock [55]. The authors were able to determine a suitable culture medium formulation to achieve the best in vitro productivity [55].

ANN is used in [56] to predict formulations of sustained-release tablets, solubility, and ratios of hydroxypropyl methylcellulose/dextrin for several tablet formulations and their accumulation and in vitro release at various sampling times. A close agreement was found between ANN-predicted and experimental findings [56] indicating the capability of ANN models for the development of formulations with suitable physicochemical properties. The trained ANN model has been able to predict optimal compositions of tablet formulations based on the proper dissolution-time profiles in vitro and release profiles in vivo [56].

An ANN and pharmacokinetic simulations were used in the design of controlled release formulations with predictable in vitro and in vivo behavior [57].

The optimized ANN model was used for the prediction of formulations based on two desired target in vitro dissolution-time profiles and two desired bioavailability profiles [57].

In [58], the authors investigate the effects of formulation factors on the in vitro release profile of diclofenac sodium from matrix tablets using the design of experiment (DOE). Formulations of diclofenac sodium tablets, with Carbopol 71G as matrix substance, were optimized by an artificial neural network [58].

In [59], the authors use the Generative Adversarial Network (GAN) framework to synthesize calcium imaging data, as a method to scale up or augment the amount of neuronal population activity data. They validated the method on artificial data with known ground-truth and synthesize data mimicking real two-photon calcium (Ca^2+^) imaging data as recorded from the primary visual cortex of a behaving mouse [59, 60]. Their results show that the GAN is capable of synthesizing realistic fluorescent calcium indicator signals similar to those imaged in the somata of neuronal populations of behaving animals. WaveGAN architecture with the Wasserstein distance training objective is used in their system development [61]. They found that Calcium GAN is able to learn the underlying distribution of the data. The authors then fitted a developed model to imaging data from the primary visual cortex of behaving mice. Importantly, it is possible to show that the statistics of the synthetic spike trains match the statistics of the recorded data.

In summary, AI-based models along with the conventional methods speed up the development and optimization of controlled release drug delivery systems [62]. They also help in the evaluation of the effects of process and formulation variables on the delivery system. The prevalent ANN in the biomedical applications could be ARTMAP, Bayesian Feedforward, Backpropagation, Hopfield, Neurofuzzy, and Resilient Propagation [63].

## 5. Conclusion and Future Work

The unique features of quantum dots which include a higher excitation coefficient broader absorption range, narrow and symmetric emission peak, and considerably good resistance to photobleaching, and many more have cemented the role of these versatile nanoparticles in the burgeoning fields of nanoscience and nanotechnology. Although quantum dots are not designed to assist everywhere, the application of quantum dots transcends beyond these artificial domains. In the future, quantum dots are going to be widely used as tools for diagnosing cancer cells. These dots can be intended for localized delivery and rate-controlled release of anticancer drugs or therapeutic agents to very specific parts of the body. Due to the property to target single organs, unlike other conventional drugs which come with unpleasant side effects, this makes them precise and more advantageous than other traditional processes like chemotherapy. These nanoparticles will also assist to categorize a wide spectrum of molecular mechanisms of various diseases as well as novel drug action mechanisms. They will be also used for observing and studying all the intracellular and extracellular activities.

In comparison to the conventional use of organic fluorophore dyes, quantum dots such as CdSe have a higher quantum yield (approximately twenty times) and better resistance to photobleaching over time, making them more suitable for long-term imaging applications. Moreover, quantum dots are excellent for multiplex bioimaging which cannot be accomplished by organic fluorophore dyes; since quantum dots have a wide absorption spectral range, their emission spectrum wavelength can be readily altered in the range of 400 nm to 4000 nm by tuning their size. Quantum dots are more cost-effective for drug delivery than gold nanoparticles, and they can also serve as carrier modules of externally guided nanobots.

However, quantum dots have also some drawbacks. In some in vitro research, degradation of quantum dots has been seen over time, which could lead to toxicities in the relevant living organisms. Quantum dots are extremely toxic and therefore must be necessarily encapsulated with a stable polymer shell. Additionally, in the core-shell quantum dot, the optical properties of the core can be obstructed by the shell and also make the regulation of the size of the quantum dots difficult.

When conjugated with targeting agents and photosensitizers, a new category of mutable multifunctional nanoparticles can be developed that might prove as an aid to diagnostics or therapy. Also, the preparation of high-quality quantum dots from compounds that are nontoxic such as carbon and silicon might reveal a superior nanocrystal which might be of paramount clinical relevance. In imaging applications and bioanalytical chemistry assays, quantum dots have recently been shown to be a promising alternative to molecular fluorophores. But these innovative trends are evident that quantum dots have a futuristic scope and will become leading fluorescent reporters in biology and medicine over the next decade. A thoughtful, vigilant, and ground-breaking approach to deal with these nifty nanoparticles might bring unparalleled miracles in the field of healthcare research.

## Figures and Tables

**Table 1:** Quantum dots in *in vitro* bioimaging [6].

Quantum dots	Purpose	Emission/size of QDs
CdSe/CdS/SiO_2_	Mouse fibroblast cell imaging	550 nm & 630 nm
CdSe/ZnS	Biological detection/sensing	1–4 nm
CdSe/ZnS/SiO_2_	Phagokinetic track imaging	554 nm & 626 nm
CdSe/ZnS	Tumor vasculature and lung endothelium imaging	<10 nm

**Table 2:** Quantum dots in *in vivo* bioimaging [6].

Quantum dots	Purpose	Emission/size of QDs
CdSe/ZnS	Tumor vasculature and lung endothelium imaging	<10 nm
CdSe/CdSe	Cancer cell lymph nodes imaging	Near infrared
CdSe/ZnS	Maltose binding protein	560

## Data Availability

The data used to support the findings of this study were obtained from the results of a literature review, and the authors do not have any new data.
